# The role of dendritic cell precursors in tumour vasculogenesis

**DOI:** 10.1038/sj.bjc.6602476

**Published:** 2005-03-22

**Authors:** G Coukos, F Benencia, R J Buckanovich, J R Conejo-Garcia

**Affiliations:** 1 Abramson Family Cancer Research Institute, University of Pennsylvania, BRBII/III, 421 Curie Blvd, Philadelphia, PA 19104, USA; 2 Center for Research on Reproduction and Women's Health, University of Pennsylvania, 1315 Biomedical Research Building II/III, 421 Curie Blvd, Philadelphia, PA 19104, USA

**Keywords:** dendritic cells, angiogenesis, vascular endothelial growth factor A

## Abstract

In this review, we discuss the recent identification *in vivo* of a population of CD11c^+^ cells exhibiting simultaneous expression of both endothelial and dendritic cell markers, termed *vascular leukocytes* (VLCs). VLCs are highly represented in human ovarian carcinomas and, depending on the milieu, can assemble into functional blood vessels or act as antigen-presenting cells. The identification of dendritic cell precursors as bipotent cells has important implications for the physiopathology and therapy of tumours. VLCs emerge as a novel therapeutic target against tumour vascularisation.

Tumours require blood supply for expansive growth. Even p53-null tumour cells, which have a reduced rate of apoptosis, die beyond an oxygen diffusion limit in the range of 150 *μ*m (Folkman, 2002). Therefore, growing tumours require the continual formation of neovasculature around which tumour cells will proliferate. These vessels are quite different from vessels of normal tissues, both at the morphological and molecular levels (Asahara *et al*, 1997; Ruoslahti, 2002). Until lately, angiogenesis, or sprouting of endothelial cells from existing vessels, was the only accepted mechanism of tumour vascularisation. More recent studies indicated that vasculogenesis, or recruitment of endothelial progenitors (EPCs) that differentiate into endothelial cells, plays an important role in the formation of tumour neovessels (Reyes *et al*, 2002). The origin of this endothelium may be either bone marrow-derived EPCs or local EPCs rooted within organs or vascular parenchyma. Initially, the only source of EPCs was thought to be a scarce haematopoietic stem-cell containing CD34^+^ population (Rafii, 2000). However, the relative contribution of bone-marrow-derived EPCs to adult neovascularisation has been lately underscored, ranging from very little to >50%. Still, Asahara *et al* (1997) and Kalka *et al* (2000) described long before a much more frequent population of EPCs expanded *in vitro* from human peripheral blood. In animal models of ischaemia, these EPCs were also incorporated into sites of active angiogenesis, suggesting that a different population of EPCs may significantly contribute to physiological or pathological vascularisation.

## ENDOTHELIAL-LIKE DIFFERENTIATION OF DENDRITIC CELLS (DCS) *IN VITRO*

It is well known that in the early embryo haematopoiesis begins in the blood islands of the yolk sac. Haematopoietic and vascular development is therefore intimately connected, apparently sharing a common mesodermal progenitor, the ‘haemangioblast’. Emerging studies have identified a population of primitive endothelial-like cells derived from human embryonic stem cells that includes bipotent cells with endothelial and haematopoietic capacity (Wang *et al*, 2004). However, until very recently, it was thought that this close association between haematopoiesis and vascularisation was restricted to the developing embryo. Already in 2000, Fernandez Pujol *et al* (2000) challenged this dogma by showing that CD14^+^ mononuclear cells cultured in the presence of angiogenic growth factors acquire the markers and morphology of endothelial-like cells. CD14 is expressed by a myeloid population with a remarkable plasticity, since it can comprise monocytes, macrophages, monocyte-derived DCs, monocyte-derived osteoclasts or DC-derived osteoclasts (Shortman and Liu, 2002; Rivollier *et al*, 2004). Similarly, Schmeisser *et al* (2001) found that a >90% pure population of CD34^−^CD14^+^CD45^+^CD31^+^ leukocytes expressed endothelial-specific markers after 2 weeks in culture under angiogenic conditions, forming endothelial-like cords and tubular structures in three dimensional Matrigel®.

Although the absence of clonal analysis and studies of proliferation cannot exclude that a tinny proportion of proliferating endothelial cells became highly represented in the cultures, these studies suggested for the first time the existence of a novel subset of cells with vasculogenic potential. In addition, a phenotypic overlap between haematopoietic DCs and endothelial cells was soon after described: Fernandez Pujol *et al* (2001) found that the same population of monocyte-derived DCs could exhibit phenotypic properties of mature DCs under inflammatory conditions, or, alternatively, behave as endothelial-like cells in an angiogenic milieu. The process of ‘endothelialisation’ was characterised by a disappearance of the leukocyte markers CD14, CD1a and CD83, and the expression of the endothelial markers von Willebrand factor (vWF), vascular endothelial growth factor receptor-2 (VEGFR-2) and VE-cadherin. Although the cells exhibited a reduced capacity to prime T cells, CD86 and major histocompatibility class II (MHC-II) were expressed at similar levels than in mature DCs. Although the purity of the culture is again an issue, monocyte-derived DCs remain in a state of low proliferation, indicating that cultured cells have a true mixed EC-dendritic cell phenotype. Another indication that DCs are the closest leukocytes to endothelial cells was provided by Harraz *et al*. By culturing a population of CD34^−^CD14^+^ monocytes under angiogenic conditions, they observed that, in the process of endothelialisation, monocytes acquire a DC phenotype, expressing markers such as endoglin and CD1a (Harraz *et al*, 2001). This *in vitro* differentiated population could be incorporated into mouse ischemic limbs, although that required coinjection with CD34^+^ cells.

More recently, other studies have confirmed the angiogenic potential of the monocyte/macrophage/dendritic/osteoclast lineage *in vitro*. By using two generally accepted criteria for the isolation of EPCs, such as uptake of acetylated low-density lipoprotein (LDL) and binding of ulex-lectin, a CD34^−^ population expressing monocyte/macrophage/dendritic markers was isolated from human peripheral blood (Rehman *et al*, 2003). Although they secreted numerous angiogenic factors, these cells did not exhibit any significant proliferation, suggesting that they may contribute to neovascularisation mainly through migration and incorporation into the vascular wall. A more challenging study has reported pluripotent capabilities for a subset of peripheral blood CD14^+^CD34^+^CD45^+^ leukocytes. These cells could be isolated and clonally expanded, giving rise not only to endothelial cells under angiogenic conditions but also to lymphocytes, macrophages, epithelial cells, and even liver cells and neurons under appropriate conditions (Zhao *et al*, 2003).

**Figure 1:**
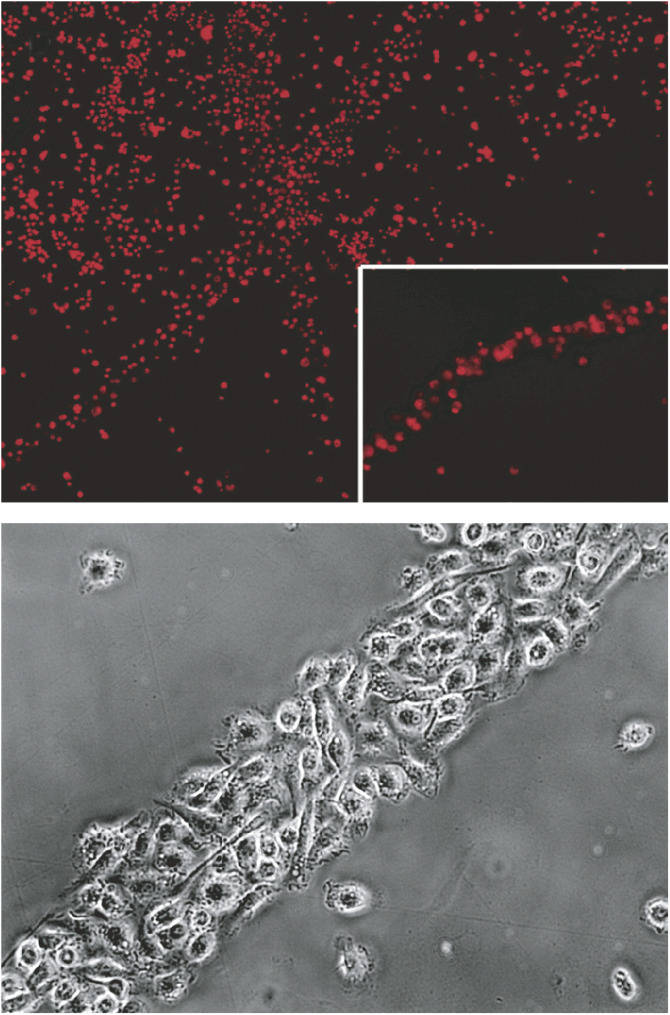
Transdifferentiation of bone marrow-derived DCs into endothelial-like cells *in vitro*. Most bone marrow-derived DCs aggregate into cord-like structures after 7 days in culture with tumour cell-conditioned media. More than 90% of them exhibited uptake of fluorescent acetylated low-density lipoprotein (top); Details of a cord composed by bone marrow-derived DCs treated with tumour cell-conditioned media for 2 weeks, which exhibits a cobblestone pattern of cell aggregation (bottom).

In an effort to understand our surprising findings in an *in vivo* model, we have dissected the process of transdifferentiation of murine bone marrow-derived DCs into cells with markers, morphology and functional properties of endothelium. We procured a >97% pure population of CD34^−^CD11c^+^ DCs by the classical method of culturing bone-marrow cells in the presence of granulocyte–macrophage colony-stimulating factor (GM-CSF). In agreement with their common use as antigen-presenting cells in immunological studies, DCs obtained in this way exhibited all the characteristics of canonical DCs, including dendritic shape, phagocytosis of apoptotic fluorescence-labelled tumour cells and maturation induced by tumour necrosis factor-alpha or bacterial lipopolysaccharide *in vitro*. They also induced antigen-specific T-cell proliferation, as assessed by ^3^H-thymidine incorporation, and secretion of interferon-gamma as well as interleukin-2 by lymphocytes. Interestingly, CD34^−^CD11c^+^ cells cultured in media conditioned by tumour cells expressing high levels of VEGF assumed a spindle-like shape within 3–5 days of culture. After 7 days, 5% of CD34^−^CD11c^+^ cells expressed CD31 by immunohistochemistry, while vWF was still not detectable. After 3 weeks, more than 80% of the cells expressed CD31 and vWF, and expressed binding sites for *Griffonia simplicifolia* B4 lectin. Incubation of CD34^−^CD11c^+^ immature DCs with tumour cell-conditioned media led to progressive alignment of single cells oriented with the same polarity, forming string-like structures (Figure 1). These grew longitudinally and recruited more cells to the sides, forming cord-like structures. Video integration showed that nearby or distant cells migrated towards alignments or cords of *vascular leukocytes* (VLCs) adhered to and finally integrated into cord structures.

By flow cytometry, most cells expressed *de novo* CD34 and upregulated CD31 within 1 week. These changes were not due to overgrowth of a small fraction of contaminating endothelial progenitors, since the rate of proliferation was very low. Instead, we theorised that our analyses captured the progressive ‘endothelial-like switch’ of CD11c^+^ DCs within the tumour-conditioned media, so we termed these CD11c^+^CD45^+^ DCs expressing CD31 and CD34, and exhibiting morphological and molecular properties of endothelial cells, VLCs. More than 90% of VLCs exhibited uptake of fluorescent acetylated LDL. Due to the phenotypic overlap between haematopoietic and endothelial cells, electron microscopy is still considered the gold standard to determine the nature of a cell. We have showed that within 3 weeks of culture with tumour cell-conditioned media, VLCs exhibited Weibel–Palade bodies and endocytic vesicles. In addition, they created intercellular junctions, organising themselves around a lumen, which are typical morphological properties of endothelial cells (Conejo-Garcia *et al*, 2004).

Remarkably, VEGF, important for the survival and proliferation of *bona fide* endothelial cells, was the critical factor for endothelialisation. Blocking VEGFR-2 (but not other VEGF receptors) with neutralising antibodies stopped the transdifferentiation process.

## VASCULAR LEUKOCYTES CONTRIBUTE TO TUMOUR VASCULARISATION

Although these *in vitro* studies revealed an unsuspected plasticity of cells from the monocyte/macrophage/dendritic/osteoclast lineage, it was not clear yet whether these changes took place *in vivo*, under physiological or pathological conditions. Moreover, although VLCs exhibit ultrastructural properties of endothelial cells, it needed to be proven that they contribute to neovascularisation.

The inhibitory role of VEGF on the normal differentiation of DC in tumours has been extensively described (Gabrilovich *et al*, 1996, 1998; Oyama *et al*, 1998; Ohm *et al*, 1999): VEGF blocks the functional maturation of DC from haematopoietic progenitor cells by blocking NF-*κ*B transcription, although it is unlikely that this inhibition alone turns these progenitors into endothelial-like cells. Confirming the critical role of VEGF on tumour immune evasion, antibodies to VEGF enhance the efficacy of cancer immunotherapy by improving endogenous dendritic cell function (Gabrilovich *et al*, 1999). Furthermore, Almand *et al* (2000) described that high levels of VEGF in blood result on decreased numbers of competent DCs and accumulation of immature haematopoietic cells. It is tantalising to speculate that these cells might exhibit an endothelial-like phenotype. Nevertheless, the angiogenic potential of DC precursors remained unsuspected until De Palma *et al* provided evidence that naturally occurring immature myeloid cells could be incorporated into blood vessels in tumours. They transduced bone-marrow progenitors with lentiviral vectors expressing green fluorescent protein under the Tie-2 promoter/enhancer, a marker thought to be endothelial-specific (De Palma *et al*, 2003). To their surprise, expression of the Tie2p/e vector identified a subset of CD45^+^CD11b^+^Tie2^+^Sca-1^+^CD34^+^ mononuclear cells that assembled a stromal structure in close association with ‘canonical’ ECs, a possible involvement in vasculogenesis.

In 2001, we viewed DCs only as critical regulators of adaptive immune responses, including those against tumours. In an attempt to create a model to study the effects of recruiting DCs intratumourally on antitumour immune response, we expressed a newly cloned *β*-defensin in an ovarian carcinoma cell line ectopically producing low or high levels of VEGF (Conejo-Garcia *et al*, 2004). *β*-Defensins are antimicrobial peptides that chemoattract immature DCs through CCR6, thus linking innate and adaptive immunity (Yang *et al*, 2004a). By recruiting DCs to tumour sites, we expected to accelerate phagocytosis and presentation of tumour antigens, thus potentiating the immune response. We indeed found a massive accumulation of CD11c^+^ cells in flank tumours and tumour ascites. CD11c is a marker mainly restricted to the DC compartment in the mouse. More than 90% of CD11c^+^CD45^+^ cells were MHC-II^+^, DEC-205^+^ and CD8*α*^+^, documenting their DC lineage, but they expressed very low levels of costimulatory molecules, and were thus identified as DC precursors. In tumours expressing physiologic levels of VEGF (comparable to those found in well vascularised organs such as kidney), expression of *β*-defensins reduced tumour growth in a flank model. However, in the presence of increased levels of VEGF, chemoattraction of CCR6^+^ DC precursors resulted in a dramatic acceleration of tumour growth and reduced survival compared to tumours with no *β*-defensin expression. Although VEGF was ectopically expressed in our model, its levels resemble those found in human tumours. Interestingly, tumours expressing *β*-defensins appeared markedly more congested and haemorrhagic compared to controls. They also exhibited a markedly different architecture, with disorganised accumulations of tumour cells, much more abundant CD31^+^ vessels and scarce stroma. When we analysed the distribution of CD11c^+^ DC precursors by immunohistochemistry, they localised to the luminal surface of capillary-like structures, admixed with CD11c^−^ cells, indicating that they had contributed to the increased vascular density.

The frequency of CD11c^+^ DC precursors in ascites increased with neoplastic progression, reinforcing the notion that CD11c^+^ cells may contribute to ovarian carcinogenesis. Similarly to the *in vitro* experiments, VEGF demonstrated to be a critical factor for the acquisition of morphological and functional characteristics of endothelial cells by DC precursors, since administration of the tyrosine kinase inhibitor SU5416 (Sugen Inc., San Francisco, CA) completely abrogated their vascular homing. Strikingly, we identified for the first time *in vivo* CD11c^+^ cells exhibiting simultaneous expression of both endothelial and leukocyte markers (VLCs). We hypothesised that our analyses within the tumour microenvironment mimicked the progressive ‘endothelial-like switch’ of CD11c^+^ leukocytes observed *in vitro*, which appeared to first acquire CD34, increase CD31 expression, and progressively lose the expression of the haematopoietic marker CD45. Tumour-infiltrating VLCs were highly represented in mouse ascites (>30% of total cells). They expressed markers common to endothelial and dendritic cells, such as CD31, vWF mRNA and VEGFR-1 (thought to be specific of endothelial cells); exhibited uptake of acetylated LDL, and bound to *Bandeiraea simplicifolia* (BS-1) lectin (our unpublished observations). Notably, they also exhibited typical endothelial-specific markers (Rafii and Lyden, 2003), such as VE-cadherin, CD146 (P1H12) and VEGFR-2. More importantly, labelled VLCs transplanted in Matrigel plugs *in vivo*, assembled into tubular structures formed by fluorescent cells, which were variably perfused with fluorescent dextran injected through the left cardiac ventricle, indicating that they had given rise to intact blood-carrying capillaries *in vivo*. Additional analysis with fluorochrome-labelled antibodies revealed that cells forming neovessels retain the expression of CD11c and CD45, confirming their leukocyte lineage. Similarly, histological analysis of solid tumour specimens revealed the presence of CD11c^+^ cells at an endothelial location. Although *bona fide* endothelial cells can also express CD11c under certain conditions (Langeggen *et al*, 2002), we also found numerous CD45^+^ vessels in all the specimens obtained with our tumour model. Of note, the same cells extracted from dissociated tumour specimens or tumour ascites were able to induce strong immune responses in naïve T cells when treated with a cocktail of inflammatory molecules, confirming their antigen-presenting cell nature.

The accumulation of immunosuppressor, immature Gr-1^+^ cells in mice with large tumour burdens has been already described (Kusmartsev *et al*, 2000), suggesting that tumours naturally recruit immature myeloid precursors to support their vascularisation. Supporting this idea, we found a population of CD45^+^CD11c^+^VE-cadherin^+^CD146^+^ VLCs in a murine lung tumour model, although less represented in the absence of ectopic expression of *β*-defensins (unpublished observations). In addition, immature myeloid Gr1^+^CD11b^+^ cells have been recently found to incorporate into tumour endothelium, acquiring endothelial cell properties (Yang *et al*, 2004b). These cells are more differentiated than classical progenitors, representing immature myeloid cells in an intermediate stage of differentiation. Of note, human tumours of different origins naturally express high levels of *β*-defensins, and ovarian cancer in particular produces other chemokines that also attract proangiogenic DCs into the tumour microenvironment, such as stroma-derived factor 1 (SDF-1) (Curiel *et al*, 2004). Therefore, it was not surprising to find that two different anti-CD45 antibodies stained tortuous, thorny vascular structures that were simultaneously positive for vascular markers in human ovarian carcinoma specimens exhibiting high levels of VEGF and *β*-defensins mRNA.

The concept that the formation of blood vessels by DCs with endothelial potential (VLCs) is not restricted to artificial tumour models, but rather constitutes a *bona fide* angiogenic mechanism promoting the progression of human tumours, is supported by the high (∼40%) proportion of VLCs systematically accumulated in ovarian carcinomas (Conejo-Garcia *et al*, 2005). Single-cell suspensions derived from freshly dispersed human ovarian carcinomas separate into three clearly isolatable fractions, according to their expression of CD45 (leukocyte) and VE-cadherin (endothelial) markers: *bona fide* leukocytes (CD45^+^VE-cadherin^−^; mostly lymphocytes according to their FSC *vs* SSC profile), *bona fide* endothelial cells (CD45^−^VE-cadherin^+^) and, VLCs (CD45^+^VE-cadherin^+^). We have identified these three populations in all specimens analysed so far (*n*=14), though in different proportions. Although the high frequency of VLCs in solid tumours may be caused by the mechanical procedure of dispersion, it is still surprising that the proportion of VLCs is comparable to that of non-VLCs.

In addition to VE-cadherin, VLCs express other endothelial markers, such as CD146 and CD34. Further characterisation of VLCs revealed that >90% are MHC-II^+^ and CD86^+^, while >86% express CD11c^+^, compatible with an immature DC phenotype. Supporting their immature character, 45% of VLCs express CD133^+^ (15–80%), a stem-cell marker with unknown function, absent in mature endothelium or committed myeloid cells. Confirming their angiogenic potential, tumour-infiltrating VLCs have the capacity to create perfusible blood vessels *in vivo* in Matrigel plugs. Experiments performed with CCR6-immunotoxins suggest that the role of VLCs to tumour vasculogenesis takes place primarily during the early stages of tumour growth. Due to their limited proliferating capacity, we propose that their angiogenic potential is mediated mainly by their recruitment to the tumour.

In addition to their capacity to become endothelial-like cells, a recent study reported the presence of CD11b^+^ leukocytes as periendothelial vascular mural cells (Rajantie *et al*, 2004). We have also found CD11c^+^ at a periendothelial location in selected specimens of our tumour model, although they always expressed CD31, a marker typically absent in classical pericytes. It is likely that these periendothelial leukocytes contribute to neovascularisation by secreting angiogenic factors. Alternatively, they may be VLCs assembling into neovessels branching out, or represent leukocytes transdifferentiating into pericytes.

## VLCS REPRESENT A NOVEL TARGET FOR IMMUNOTHERAPY

The potential to inhibit tumour growth by targeting VLCs is illustrated by our studies with CCR6-immunotoxins: Tumours injected in the presence of anti-CCR6 antibodies bound to saporin, a ribosomal-inactivating protein, grew 2.6-fold less than tumours injected with an isotype control antibody. Since decrease in tumour growth was associated with a reduced number of CD11c^+^ infiltrating cells, VLCs emerge as novel therapeutic targets. Antibodies directed against specific antigens and conjugated to toxins may contribute to block tumour vascularisation. On the other hand, we have found a higher presence of VLCs in the tumour periphery, suggesting that, as the tumour grows, VLCs stick preferentially around the tumour. Therefore, VLCs appear as optimal candidates for targeted delivery of therapeutic agents as ‘Trojan Horses’. For instance, adoptive therapy of VLCs expressing suicidal genes with bystander effect may help to collapse tumour neovessels, thus triggering both necrosis and antitumour immune responses. Alternatively, transduction of VLCs with vectors expressing immune factors under the control of endothelial-specific promoters may negate their angiogenic potential once they have been incorporated into the vessel wall, delivering inflammatory cytokines into the tumour microenvironment.

## TRANSDIFFERENTIATION IN THE TUMOUR MICROENVIRONMENT OR RECRUITMENT AS DUAL CELLS?

**Figure 2:**
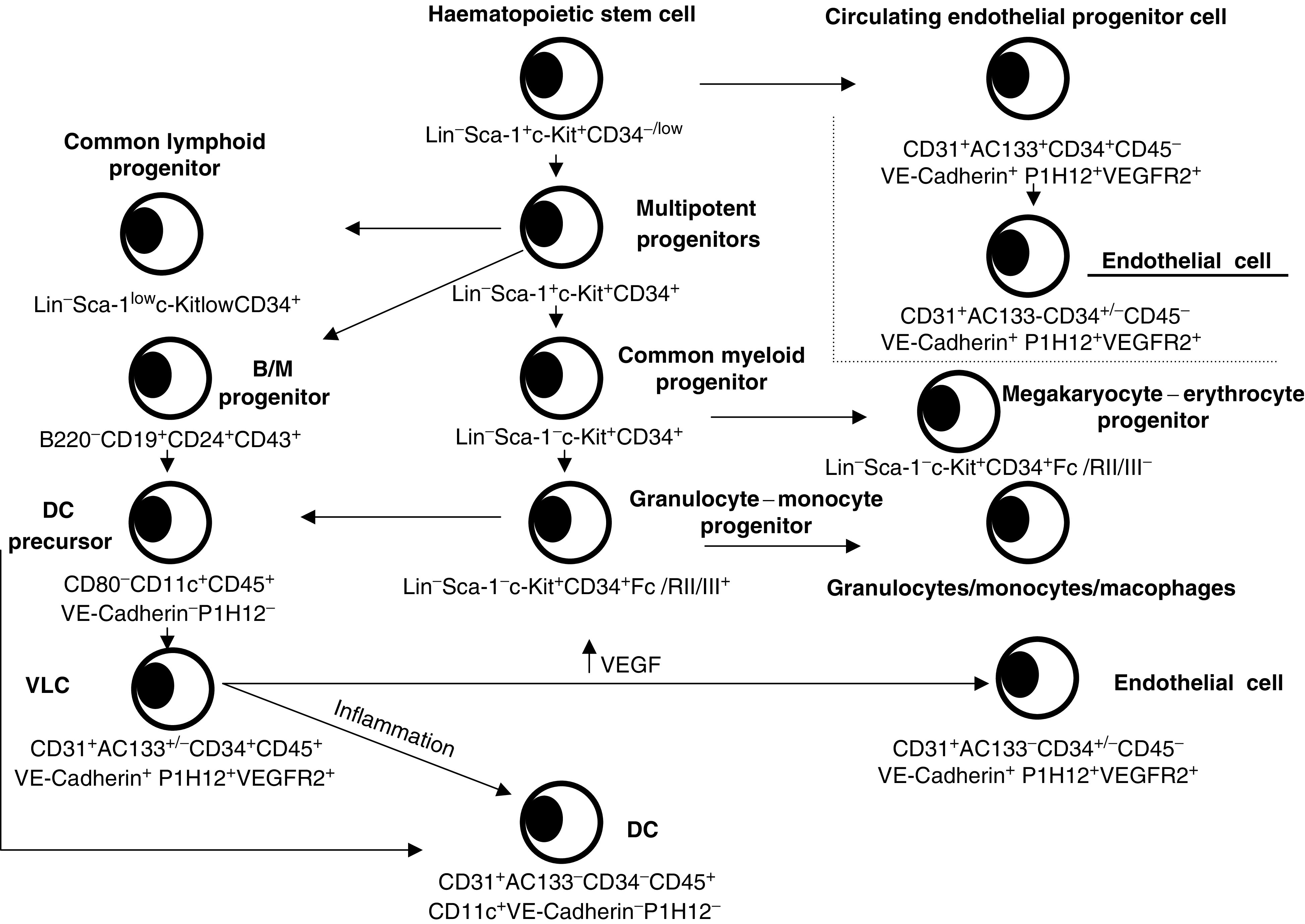
Hypothetical scheme of the transdifferentiation of DC precursors into endothelial-like vascular leukocytes (VLCs) in mouse tumours. DC: dendritic cells; MΦ: macrophage.

An important question that remains unresolved is whether VLCs are directly recruited as cells with ‘haemangioblast’ properties, or rather as leukocytes in a low stage of differentiation that turn into dual cells in the tumour microenvironment (Figure 2). Several studies conducted by our group support the later hypothesis: First, we have not found CD45^+^CD11c^+^VE-cadherin^+^CD146^+^ cells in peripheral blood of patients with ovarian carcinoma. Similarly, we have detected VLCs in different bone-marrow and lymphoid organs of tumour-bearing mice at very low frequencies (<0.2%). Second, we have demonstrated that the tumour microenvironment induces DC to differentiate *in vivo* into endothelial-like cells. We injected BM-derived DCs labelled with a fluorochrome subcutaneously around tumours overexpressing VEGF. At 3 days postinjection, fluorescent DCs microdissected by laser-capture exhibited a 2.6- and 6.1-fold upregulation of *CD31* and *VE-cadherin*, respectively, compared to preinjection levels, while 7 days postinjection a 60- and 22-fold upregulation was noted, respectively, by real-time quantitative PCR. Furthermore, *VEGFR-2* was upregulated 20 fold. Collectively, these data suggest that the tumour microenvironment can induce the endothelialisation of immature DCs, thus mimicking the transdifferentiation process observed *in vitro*. In addition, we have observed that labelled bone marrow-derived DCs assemble into neovessels in Matrigel plugs, a process that can be blocked by addition of neutralising antibodies against VEGFR-2. However, although they are generally used for every kind of immunological studies, it is still possible that bone marrow-derived DCs do not belong to the DC type naturally found in tumours and possess more plasticity.

## CONCLUDING REMARKS

The identification of DCs or DC precursors as bipotent cells has important implications for the physiopathology and therapy of tumours. As VLCs are an important host cell population affecting tumour growth, important concerns emerge regarding the use of intratumoural DC inoculation as a means for tumour vaccination in the human. Similarly, if VLCs are inefficient APCs, what is their exact role in immune tolerance of tumours? It is also intriguing to hypothesise that DC precursors might turn into *lymphatic endothelial-like* cells under the influence of VEGF-C, thus contributing to tumour metastasis. Finally, important questions arise on numerous issues. Which is the relative contribution of VLCs to tumour vascularisation? At which point of tumour development is it more relevant? Is the contribution of VLCs to vasculogenesis organ-dependent or affects different tumours in similar proportions? And most importantly, are VLCs contributing to physiologic vascularisation? The existing evidence that endothelium increases APC capabilities and specialised endothelium may participate in maintenance of peripheral tolerance raises intriguing questions on the ontology of endothelial cells in normal tissues and the relationship of angiogenesis and peripheral tolerance.
